# Augmented reality and mixed reality for healthcare education beyond surgery: an integrative review

**DOI:** 10.5116/ijme.5e01.eb1a

**Published:** 2020-01-18

**Authors:** Jaris Gerup, Camilla B. Soerensen, Peter Dieckmann

**Affiliations:** 1 School of Medical Sciences, University of Copenhagen, Denmark; 2 Department of Pediatrics, Herlev and Gentofte Hospital, Denmark; 3 Copenhagen Academy of Medical Education and Simulation (CAMES), Center for Human Resources, Herlev and Gentofte Hospital, Denmark

**Keywords:** Augmented reality, mixed reality, healthcare education, medicine, integrative review

## Abstract

**Objectives:**

This study aimed to review and synthesize the current research and state of augmented reality (AR), mixed reality (MR) and the applications developed for healthcare education beyond surgery.

**Methods:**

An integrative review was conducted on all relevant material, drawing on different data sources, including the databases of PubMed, PsycINFO, and ERIC from January 2013 till September 2018. Inductive content analysis and qualitative synthesis were performed. Additionally, the quality of the studies was assessed with different structured tools.

**Results:**

Twenty-six studies were included. Studies based on both AR and MR involved established applications in 27% of all cases (n=6), the rest being prototypes. The most frequently studied subjects were related to anatomy and anesthesia (n=13). All studies showed several healthcare educational benefits of AR and MR, significantly outperforming traditional learning approaches in 11 studies examining various outcomes. Studies had a low-to-medium quality overall with a MERSQI mean of 12.26 (SD=2.63), while the single qualitative study had high quality.

**Conclusions:**

This review suggests the progress of learning approaches based on AR and MR for various medical subjects while moving the research base away from feasibility studies on prototypes. Yet, lacking validity of study conclusions, heterogeneity of research designs and widely varied reporting challenges transferability of the findings in the studies included in the review. Future studies should examine suitable research designs and instructional objectives achievable by AR and MR-based applications to strengthen the evidence base, making it relevant for medical educators and institutions to apply the technologies.

## Introduction

The integration of digital strategies has brought healthcare education to a paradigm shift, now reflected in many educational curricula.^1^ Modern teaching curricula aim to educate trainees efficiently in safe environments to establish transferability into the clinical context. Augmented reality (AR) and mixed reality (MR) have long been expected to be disruptive technologies, with potential uses in medical education, training, surgical planning and to guide complex procedures.^2^ While virtual reality (VR) has mainly led the way for the implementation of the display technologies, it is criticized for several limitations.^3^^,^^4^ The term display technologies will hereafter be used to refer to AR and MR although it in principle also covers VR. The latter, however, is beyond the scope of this review.

AR describes display-based systems that combine real and virtual imagery, which are interactive in real-time and register the real-world environment to be augmented by virtual imagery.^5^  The visual display technology augments the physical environment by especially two principal manifestations: See-through (transparent) head-mounted display and non-immersive monitor-based video (window on the world). ^6^ AR systems are based on the combination of the physical and the virtual environment. On the contrary, in VR systems the participant is totally immersed in a completely virtual one.

MR is defined as the merging of real and virtual worlds and can be seen as a larger class of technologies covering the display environment of AR and augmented virtuality (AV).^7^ Where virtual information augments the real view in AR, real-world information augments the virtual scene in AV. The external inputs providing real-world context are also seen in VR but were classified as MR in this review. The term of MR was included to embrace new technology labeled as MR, that tries to define a clear distinction between AR and MR, even if there is none.^8^

The abilities to provide situated and authentic experience connected with the real environment, enhance interaction between the physical and virtual content, while preserving a feeling of presence explains the growing expectations that AR and MR may be suitable for healthcare education in various contexts.^9^

Concerning healthcare education, the process of teaching, learning and training with an ongoing integration of knowledge, experience, skills and responsibility qualifies an individual to practice medicine.^10^ Looking into medical education, several authors request to eliminate outdated, inefficient, and passive learning approaches and start to embrace these newer methodologies of learning.^11^ Surgeons have historically always been quick to adapt to new technology developing new treatment and learning methodologies, while physicians were rather more tardy.^12^ Today most studies on display technologies stem from surgery. In an integrative review on AR in healthcare education from 2014, surgical studies accounted for 64% (n=16) of the studies included.^13^ A recent systematic review on AR for the surgeon clarifies the current lack of systematic reviews for physicians and ultimately educators within the field of medicine.^14^ Many internists and other medical specialists do no longer diagnose and treat illnesses using only their knowledge of pathophysiology and pharmacology.^15^  Today, many physicians have taken up procedures and surgical treatment initiatives by operation or manipulation defined as the use of hands to produce the desired movement or therapeutic effect in part of the body.^16^ Nevertheless, medicine consists essentially of non-surgical treatment, procedures and other approaches of diagnostics and prevention of disease that need to be taught, learned and trained with an ongoing evaluation of adaptations. AR and MR may effectively help medical educators achieve such instructional objectives for medical education as it is being used for surgical training.

According to the review by Zhu and colleagues, publications in the field of AR increased significantly in 2008.^13^ Now, ten years after that publication outbreak, a new review is warranted. To the best of our knowledge, current reviews on AR and MR have not specifically studied applications for medical subjects in healthcare education. Most papers predominantly include surgical studies and only a few focused on AR in either otolaryngology or medical training.^1^^,^^3^^,^^4^^,^^9^^,^^13^^,^^17^ Currently, no adequate reviews are available that uncover the educational profile of both AR and MR-based applications across different medical specialties, subjects and target groups.

Our aim of this integrative review was to investigate the current research and state of AR and MR-based applications for healthcare education beyond surgery, providing an overview of the findings, strengths and weaknesses of the reported studies.

## Methods

We chose to conduct an integrative review, given that previous reviews showed only a few studies relevant for the current scope.^3^^,^^4^^,^^13^^,^^17^ This is thought to be the broadest type of review as it allows the inclusion of various research designs and information sources.^18^ The method also integrates a process of quality assessment of the studies included that may qualify the integrative review for recommending practice and answering complex search questions.^19^^,^^20^ The digital databases of PubMed, PsycINFO and ERIC were searched. The journal of Medical Teacher was hand-searched. Ted Talks and podcasts on the iTunes Podcast app were included, acknowledging the increasing importance of “new media”.^21^^,^^22^ Studies published between January 2013 and September 2018 were included. Relevant word groups, combinations and open-ended terms used for the search were: “Augmented reality OR mixed reality” AND “medicine OR medical OR healthcare” AND “educat* OR simulat* OR train* OR learn*”. We did not implement any filter of ‘NOT virtual reality OR surgery’ in our search string to avoid missing relevant studies examining non-surgical elements despite being termed as a surgical study.

### Eligibility criteria

The selection process was done according to three overall criteria regarding research, focus on technology and content. According to the criterion of research studies were included if they described 1) a goal or research question, 2) an appropriate study design, 3) data collection and analysis methods and 4) the discussion of results.  Research articles were excluded if they 1) neither described goal nor research question, 2) were review papers and 3) were focused on system descriptions without evaluation or other data. Table 1 provides the inclusion and exclusion criteria for the study.

### Study selection

All abstracts were read by JG, who assessed whether they met the inclusion criteria. In case of doubt, JG discussed the inclusion of studies with the other authors. All duplicates were removed.

### Data extraction and synthesis

Study characteristics and information of all articles were extracted and described by JG. Characteristics were authors, study aim, subject of healthcare education, design, participants, outcome measures, results, application/technologies, training time and display system. Content analysis was used to describe the study designs and to inductively identify the strengths and weaknesses of AR and MR as described by the studies included.

**Table 1:** Inclusion and exclusion

Criterion	Inclusion criteria	Exclusion criteria
Research	Goal or research question described	Neither goal nor research question described
	Study design described and appropriate	Review papers
	Data collection and analysis methods were described	System description without data evaluation
	Results were described and discussed	
Focus on technology	Combination of real and virtual environments	Used augmented or mixed reality in name but investigated only virtual reality
	Interactive in real-time	
	Real or perceived registration in 2D or 3D	
Content	Healthcare education	Education without medicine or only surgical focus
	Medical education	Medicine without education or only treatment or rehabilitation focus
		Patient education related to treatment
		Dentistry, veterinary medicine or other fields of education

### Quality assessment

The methodological quality of quantitative and mixed methods studies was evaluated with the Medical Education Research Study Quality Instrument (MERSQI).^23^ This 10-item instrument has been thoroughly assessed and evaluated for its correlation with other assessment tools for research quality.^24^ MERSQI covers six domains of studies: Study design, sampling, type of data, the validity of evaluation instrument, data analysis and outcome. All domains assign 0-3 points valuing the study to a final score between 0 and 18, the larger number indicating better study quality. The score will be presented as mean, standard deviation (SD) and range in parentheses. Each study was scored at the highest possible level. If a study reported more than one outcome, the rating for the highest outcome score was recorded not differentiating between primary or secondary outcome.

The quality assessment of all studies was done by JG. In addition, to assess the quality of JG evaluation, a level of approximately 20% of the studies were randomly selected for assessment by co-authors and independently evaluated by at least two authors. We computed the intraclass correlation coefficient (ICC) to calculate the inter-rater reliability (IRR) between all authors.

The methodological quality of qualitative studies was evaluated with a 12-item grid for Appraising Qualitative Research Articles in Medical Education that was converted into a quality assessment tool (AQRAME) by the authors of this review.^25^ The instrument covers five domains: Introduction, methods, results, discussion and conclusion. The domain of methods assigns 0-5 points and the conclusion domain only assigns 0-1 point, while the three remaining domains assign 0-2 points. It includes a score range between 0 and 12 points, with a larger number indicating better study quality. A score of 0.5 was given in case of an unclear answer of neither yes nor no. The score will be presented as mean, SD and range in parentheses.

An overall quality assessment tool was developed for rating all included studies regardless of their methodological design, assigning a figure of 1 to 7, with the larger number indicating better study quality. This was introduced to challenge the relative judgements of the MERSQI and AQRAME, acknowledging that different research questions inherently require different study designs. The appraisal was based on the need to be explicit about the role and assessment of the researcher in qualitative research.^26^ For studies with mixed-method designs, we applied the MERSQI tool only, rating the quantitative parts of the study.

## Results

**Figure 1:**
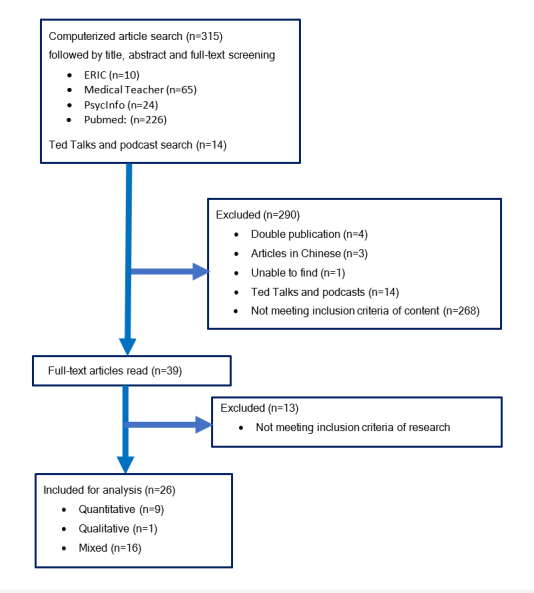
Selection process of studies

Out of the 315 papers initially identified, four duplicates were removed, three articles in Chinese excluded, and one article could not be retrieved. No reporting of research was found in 14 Ted Talks and iTunes podcasts. Three hundred seven publications were screened and 281 excluded as they did not meet the inclusion criteria. Study subjects related to nasogastric tube insertion, facet joint injection, catheterization or needle guidance were interpreted to clinically related to medicine as a practice of diagnosis and so these studies were classified to fulfill the inclusion criteria. One study focusing on resection planning was included and categorized as preoperative visualization.^27^ However, needle insertion itself was interpreted not to produce a desired movement or therapeutic effect in part of the body and not classified as a surgical procedure. This resulted in a total of 26 studies being included in the integrative review. The flow chart of publications selected for inclusion in this integrative review is displayed in Figure 1.

### Study characteristics

The studies applied AR and MR primarily by integrating the display technologies into knowledge platforms and guidance systems for simulator practice. Some studies offered feedback in the endeavor of a skill or a field of knowledge, while others provided an immersion into scenarios and remote assessment-training for telemedicine. The display technologies showed the ability to stimulate the learning process and support the learner for several competencies:

To understand spatial relationships and construct mental 3D models of anatomy with the help or without 2D imaging. To acquire cognitive-psychomotor abilities, prolong learning retention, experience student-centered motivation and obtain flexibility to learn anytime and anywhere in their own pace and style. Furthermore, the studies suggested that AR and MR could complement practice in safe simulation environments contributing to patient safety and a higher degree of confidence (See Appendix 1 – “Summary of results”).

### Technical specifications

The majority of studies (n=22) examined an actual application of AR.^28^^-^^49^ The rest (n=4) investigated an application based on MR.^27^^,^^50^^-^^52^Six applications developed by companies were reported in 10 studies.^30^^,^^31^^,^^37^^,^^39^^,^^40^^,^^43^^,^^47^^,^^48^^,^^50^^,^^51^ The remaining studies (n=16) involved self-developed applications primarily developed at universities and hospitals.

Mobile device-based (tablets and smartphones) applications were used in nine studies.^33^^,^^35^^,^^37^^,^^39^^,^^41^^,^^42^^,^^47^^-^^49^ Of these two thirds (n=6) involved camera and marker-based recognition, and three studies did not report any further on the applications developed.^41^^,^^47^^,^^48^ Eight studies implemented head-mounted display.^27^^,^^28^^,^^38^^,^^40^^,^^43^^-^^46^ Two studies utilized the same head-mounted display.^40^^,^^43^ The head-mounted display-integrated applications had marker-based recognition in four of the studies.^28^^,^^40^^,^^43^^,^^44^ One study recognized the hands and gestures of a mentor projecting these into in the trainee’s display.^46^ Two studies implemented a foot pedal to interact with the application.^27^^,^^38^ For one study this included toggling between AR and MR-mode.^27^ Computers were used in 11 studies.^30^^,^^31^^,^^34^^,^^36^^,^^38^^,^^40^^,^^43^^,^^46^^,^^50^^-^^52^These delivered the computing power for head-mounted display-based applications in four studies. ^38^^,^^40^^,^^43^^,^^46^ One computer-based application had marker-based recognition.^36^ Seven studies were sensor-based.^30^^,^^31^^,^^34^^,^^46^^,^^50^^-^^52^Two studies recognized landmarks of the user’s body.^30^^,^^31^ Four studies recognized a virtual model registered with a phantom characterized as MR.^27^^,^^50^^-^^52^Eleven studies reported using external cameras and tracking devices.^27^^,^^28^^,^^31^^,^^32^^,^^34^^,^^36^^,^^44^^,^^50^^-^^52^Two studies used applications based on projectors, one recognizing markers on a phantom, and one projecting images directly onto a phantom without using a tracking device.^29^^,^^51^

### Methodological quality

In the included 26 studies, nine were solely quantitative, 16 were mixed research methods and one was qualitative. Based on rating comparisons of the approximately 20% (n=5) randomly selected papers, the authors’ agreed to use the ratings by JG for MERSQI, AQRAME and the overall score for the remaining papers. The average total MERSQI score of the 25 quantitative and mixed methods studies was mean 12.26, SD=2.63 (7-15.5). The ICC between all raters were computed to IRR=.50 for the MERSQI overall score, which corresponds to a moderate reliability.^53^ Nearly one-third of all studies (n=8) either had no evaluation tool or did not report any validity of the instrument used.^28^^-^^35^

The qualitative study involved semi-structured face-to-face interviews that explored the needs and challenges of applying AR for healthcare education. The study demonstrated a detailed clarity and rigor according to the individual AQRAME score of all three authors corresponding to 12 (JG), 11.5 (CBS), and 12 (PD). As there was only one qualitative study, we did not report any IRR for the AQRAME overall score.

The mean average overall quality score of all studies was 4.08, SD=1.65 (1-7) with an adjusted ICC equaling IRR=.429 also corresponding to a moderate reliability.^53^ The scores of the individual studies and the study characteristics are reported in Appendix 1.

### Strengths and weaknesses of AR and MR

Three themes were inductively identified indicating the strengths and weaknesses of AR and MR in healthcare education beyond surgery.

### Strengths

#### Implemented across various subjects for learner types of all levels spanning different sectors

The most frequently studied subjects of healthcare education were found within anatomy (n=6) and anesthesia (n=7), the ladder represented by four studies focusing on central vein catheterization.^29^^,^^38^^,^^44^^,^^52^ Study participants were divided into 12 different categories: Pre-medical, medical, nursing, and health science students, novices, residents, fellows and established clinicians of different specialties, technicians, non-clinicians, non-specified participants and managers. The mean number of participants was 77.1, SD=170.6 (1-880) since the sample size was set to one in a study that did not report or specify the study participants.^33^ The distribution of studies across subjects of healthcare education related to the number of participants enrolled is described in Appendix 2.

#### The rich diversity of research and outcome focus

A total of six proof-of-concept, pilot or user studies sought to introduce an application or assess initial validity.^28^^,^^29^^,^^33^^-^^35^^,^^47^Eight studies focused on evaluating training by an application for strengthening the validity of the construct.^30^^,^^37^^,^^39^^,^^40^^,^^42^^,^^43^^,^^50^^,^^51^ The remaining studies (n=12) focused on the application-based assessment of a specific skill or procedure, eventually correlating the performance to other outcomes such as cognitive load.^27^^,^^31^^,^^36^^,^^38^^,^^41^^,^^44^^-^^46^^,^^48^^,^^49^^,^^51^^,^^52^ Technical test outcomes were reported in 17 studies and concerned primarily needle insertion in terms of accuracy and precision (n=11).^27^^-^^29^^,^^31^^,^^33^^,^^40^^,^^43^^,^^44^^,^^50^^-^^52^ The secondly most reported technical test outcome concerned procedure time (n=9).^27^^,^^29^^,^^38^^,^^43^^,^^44^^,^^46^^,^^50^^-^^52^ Nineteen studies investigated learning experience and user acceptance based on especially Likert scales.^30^^-^^32^^,^^34^^-^^42^^,^^44^^-^^49^^,^^52^ Other questionnaire-based outcomes were cognitive load, stress response, adverse health effects and ergonomics.^38^^,^^39^^,^^41^^,^^44^^-^^46^ Knowledge tests were examined in combination with questionnaire-based outcomes in six studies.^36^^,^^37^^,^^39^^,^^41^^,^^42^^,^^49^ One study included an observational method to determine learning behavior.^49^

#### Growing evidence for improving learning

In 11 studies AR and MR were claimed to significantly improve the learning process or part-tasks associated in all or in the majority of outcome measures.^27^^,^^29^^,^^36^^,^^37^^,^^39^^,^^40^^,^^43^^,^^48^^-^^50^^,^^52^ Four out of six studies examining the acquisition of anatomy knowledge reported significantly improved learning.^36^^,^^37^^,^^39^^,^^49^ Significant positive findings were found in six of 11 studies concerning skill training of needle insertion favoring both students and established clinicians.^27^^,^^29^^,^^40^^,^^43^^,^^50^^,^^52^ Procedure time was significantly reduced in three of nine studies.^27^^,^^29^^,^^52^ Examining different questionnaire-based aspects of the learning experience and user acceptance four of 19 studies demonstrated significant positive findings advocating the usability

of the display technologies.^36^^,^^37^^,^^39^^,^^48^ Fifteen studies found no significant positive results but all suggested the AR and MR-based applications may outperform traditional learning approaches within the involved subjects of healthcare education.^28^^,^^30^^-^^35^^,^^38^^,^^41^^,^^42^^,^^44^^-^^47^^,^^51^ Other promising learning factors facilitated by the display technologies were related to visualization, directing attention, intrinsic benefits of motivation, physical interaction activating kinesthetic schemes, patient safety, skill retention, simulation confidence related to transferability, mobile learning and using oneself as a learning object.^39^^,^^41^^,^^42^^,^^45^^,^^49^^,^^51^

### Weaknesses

#### Reporting of prototypes, technological limitations and poor ergonomics

Sixteen studies presented a prototype, typically as preliminary feasibility studies lacking to report adequately on the educational impact of the prototype tested.^27^^-^^29^^,^^32^^-^^36^^,^^38^^,^^41^^,^^42^^,^^44^^-^^46^^,^^49^^,^^52^ Ten studies were conducted on one of six established applications.^30^^,^^31^^,^^37^^,^^39^^,^^40^^,^^43^^,^^47^^,^^48^^,^^50^^,^^51^ The studies of head-mounted display-based applications (n=8) addressed technological limitations related to limited computing power, occlusion of the user’s field of view and poor ergonomics by head-mounted displays being tethered to workstations and when wearing glasses underneath.^27^^,^^44^^,^^46^

#### Shortcomings of the study designs for transferability

Four studies were designed as a single group user study only, making strong conclusions difficult.^31^^-^^33^^,^^35^ Twenty-two studies used a group design or comparison, of which the most (n=17) compared two groups.^27^^-^^30^^,^^34^^,^^36^^,^^38^^-^^40^^,^^42^^,^^44^^,^^45^^,^^47^^-^^51^Only two studies did not compare AR or MR with another media corresponding to lectures, books, video, virtual reality, mobile devices, conventional training platforms, and telemedical full-setup.^28^^,^^34^  Two studies compared the media of mobile devices after having provided AR content to one of the groups.^41^^,^^42^ Five studies encompassed three groups.^37^^,^^41^^,^^43^^,^^46^^,^^52^ Two of the two-group studies used a cross-over design.^29^^,^^30^ No study involved patients in an authentic context, but two studies included patient data.^27^^,^^32^

#### Lacking evidence for improving learning

Eight studies reported descriptive frequencies of self-reported evaluations and measures without any statistical analysis of significance.^28^^,^^30^^-^^35^^,^^47^Seven studies claimed the display technologies offered no significant impact for improving learning in all or in the majority of outcome measures. ^38^^,^^41^^,^^42^^,^^44^^-^^46^^,^^51^ The two studies that compared AR within the same media of mobile devices found no significant difference in any of the outcome measures.^41^^,^^42^ Only a single study presented a significant negative finding of prolonged completion time of an ultrasound examination in the AR group.^46^ Potentially conflicting factors were addressed in terms of visual misperception, media or technology enthusiasm-based motivation, negation of patient discomfort related to patient safety, and missing translation of performance from simulation to clinical setting.^27^^,^^41^^,^^50^^,^^51^

## Discussion

Virtual augmentation and guidance of AR and MR are increasingly used in applications for medical subjects of healthcare education these years. The quality of the existing studies and applications including the educational benefits of the display technologies remain unclear at the moment.

We reviewed the current research and state of AR and MR-based applications for healthcare education in medical disciplines beyond surgery. Our integrative review identified 26 original studies examining various applications of both display technologies. The applications were found to measure numerous outcomes related to the learning process, acquisition of knowledge and skill training while providing feedback on patient care-related outcomes such as complication rates, insertion time and needle path related to tissue damage. This differs greatly from the findings of a systematic review by Barsom and colleagues on applications for medical training for professionals, in which none were developed to measure the prevention of errors for the interest of patient safety.^4^

Our work revealed an increased emergence of established applications corresponding to 27% (n=6) investigated in 10 studies against 16 prototypes. A prior review by Zhu and colleagues only found one established application for laparoscopic colorectal surgery.^13^ In the same review, the authors found the application designs lacking guidance by learning theories only resting on traditional learning strategies. We observed that the applications of AR and MR still have not exploited the integration of learning theories and strategies into their design. Still, the increased number of established applications is a step towards turning the research base away from feasibility studies examining prototypes.

We conclude that the studies overall were of low-to-medium quality. This is consistent with the low to modest strength of evidence level reported in previous systematic reviews.^4^^,^^17^The single qualitative study was found to be of high quality in terms of clarity and rigor, while the relative judgement of the overall quality was found to be of a low-to-medium quality. The greatest limitation across the pool of studies noted in nearly one-third of all studies (n=8) was either the utter lack or poor reporting of the validity of the evaluation instruments indirectly providing the evidence base for the study findings. Additionally, the statistical analyses reported incomplete results or were unclearly interpreted. Shortcomings of the reviewed studies further included heterogeneity of research designs, unstandardized outcome measures and wide variation in details given. Widespread heterogeneity among studies is stated to be one of the greatest challenges of quantitatively synthesizing research evidence.^54^ At the same time, an outspoken concern argues that media-comparative studies in learning are virtually useless and not valid for comparison.^55^ From this perspective, the studies failed to determine which media or technologies were best for healthcare education but rather informed practice with the specific application. These limitations are general for much education research but may be especially pronounced for research in the nexus of learning and technology.^56^ Nevertheless, we did not exclude studies based on their quality due to our aim of providing an overview of the strengths and weaknesses of all relevant research in AR and MR for healthcare education beyond surgery during the past half-decade.

### Limitations and recommendations for future studies

To our knowledge, this is the first integrative review of AR and MR solely focusing on medical subjects of healthcare education. Three articles in Chinese were not included, meaning that we possibly excluded relevant knowledge. Moreover, we may have missed relevant research either published or not published in technical journals as our main focus was on databases for healthcare and education. Our finding that all included studies suggested or reported significant positive findings should be interpreted with caution since publication bias cannot be excluded. We tried to minimize the drop-out of relevant material by including unpublished work from new online sources such as TED Talks and the podcast media of iTunes. There was a contentious issue of the designs and presentations of these varying too extensively without enhancing the quality and usefulness of the review. Our study abstained from addressing the educational profile of AV compared to AR both being encompassed by MR. This could not be done due to a low number of studies measuring AV-based learning, possibly related to the impaired technologic and conceptual understanding of MR across the research field and industry. The quality of the included studies was assessed with the MERSQI scale, which revealed inconsistencies across a few domains in the process of rating. This was mainly due to missing information in the reviewed studies as well as a lack of clarity in the MERSQI guidelines. Though moderate reliability was found between all raters in the MERSQI and the overall quality assessment tool, one could argue that the sample size of the rating corresponding to approximately 20% (n=5) of the studies either hinders or disallows reliable calculations beyond descriptive analysis. Finally, the self-developed assessment tool of AQRAME has not been validated for quality scoring qualitative research despite relying on a known 12-item grid for quality appraisal. This tool was introduced since we were not aware of any validated evaluation instruments for quality assessment of qualitative research in healthcare education.

A variety of applications for subjects of healthcare education beyond surgery have been developed, and their benefits were supported by this integrative review. We expect that more research will be done on the field as more institutions will explore and apply applications based on AR and MR in the future. Randomized controlled trials should continuously be organized for evaluating clinical performance and patient-care related outcomes. Specifically, the actual effects on real patients and physician behaviors towards patients in a real context are yet to be elucidated. We recommend future studies to justify and validate metrics and report the reliability of measures for higher-quality evaluations. Established guidelines and recommendations for high-quality research formulating joint standards could promote the adoption of the display technologies and facilitate exchange among researchers, educators and developers with widely different experiences and approaches.^57^

Similar to the words of David A. Cook, professor of medicine and medical education, we suggest placing more emphasis on the ‘How’ and ‘When’ to use AR and MR-based learning and to focus less on ‘Whether’.^55^ Answering these questions researchers, educators and developers should share and evaluate the instructional design and learning theory-based methods while looking into effective use of simulation, and integration of the display technologies within and between institutions. Eventually, this could also provide an understanding of learning concepts revealed from the included studies involving intrinsic benefits of motivation, physical interaction activating kinesthetic schemes, skill retention, transferability of simulation confidence, mobile learning and using oneself as a learning object. By defining instructional objectives beforehand, the display technologies should be used only when it could refine or even replace training programs and curricula.

With that being said partially immersive environments such as AR and MR may offer unique qualities for specifically, assessment and training procedural strategies integrating real patient data and without breaching patient safety. By using non-invasive sensors for imaging, the display technologies could complement the established imaging technologies of MRI, CT scan and ultrasound for monitoring of technical performance with an objective-comparative function as observed in our review.^27^^,^^29^^,^^50^ To tap the full potential of the display technologies, the study and application design must be based on a throughout investigation of the educational context, learner types and learning objectives whether the latter being cognitive, technical, or non-technical such as measuring situational awareness, communication, or stress coping.

## Conclusions

This review reports the current state of AR and MR-based applications for healthcare education beyond surgery. Studies based on both display technologies across various specialties and subjects states an increased number of established applications moving the research base away from feasibility studies on prototypes. All included studies suggested various healthcare educational benefits by the display technologies which significantly outperformed traditional learning approaches in 11 studies, specifically regarding the acquisition of anatomy knowledge and needle insertion skills. Yet, this review identifies multiple shortcomings of the studies. Study quality was low-to-medium especially due to lacking validity of the evaluation instruments, heterogeneity of research designs and widely varied reporting. Future studies are thus needed for researchers, educators and developers to build an evidence base defining suitable research designs and instructional objectives achievable by AR and MR-based applications, for these to complement conventional learning, curricula, and conduct a transformation in healthcare education.

### Acknowledgements

We would like to thank for financial support by the institutional funds of the Copenhagen Academy of Medical Education and Simulation (CAMES), and valuable feedback by the employees of the academy.

### Conflict of Interest

PD holds a professorship with the University of Stavanger, Norway that is supported unconditionally by a grant from the Laerdal Foundation in Norway.

## Supplementary material

Supplementary file 1 Appendix 1. Study characteristics including quality scores

Supplementary file 2 Appendix 2. Distribution of studies across medical specialty or health science, number of studies, and participants enrolled according to number of studies
