# Supplementary material for: Augmented reality and mixed reality for healthcare education beyond surgery: an integrative review
Source: Int J Med Educ. 2020 Jan 18;11:1–18. doi: 10.5116/ijme.5e01.eb1a (PMC7246121; doi:10.5116/ijme.5e01.eb1a)
Supplement: Supplementary file 1 — Appendix 1. Study characteristics including quality scores [file ijme-11-1-S1.pdf]

Appendix 1.

Study characteristics including quality scores

Study of a Quantitative Method	Study Aim (Subjects of Healthcare Education)	Design (Participants)	Outcome Measures	Summary of Results	Application/ Technologies (Training time)	Display System	MERSQI Score (18)	Overall Rating (7)
Abhari et al. (2015)	Evaluation of an HMD-based guidance system compared with three planning environments<br><br>(Resection planning of brain tumour from images and head phantom)	Single-group posttest (Study 1 and 2)<br>(10 novices/non-clinicians)<br><br>Two-group non-randomized comparison (Study 3)<br>(7 clinicians and 14 novices/non-clinicians)	Test:<br>1) Difference in points of entry<br>2) Deviation between angles of surgical path<br>3) Accuracy<br>4) Response time<br>5) Index of performance	AR/MR significantly improved non-clinicians' performance (p<.01) compared to conventional planning environments<br>(Study 1 and 2)<br>AR/MR guidance significantly reduced the time of the task performed by clinicians (p<.05)<br>(Study 3)	Self-developed for HMD with tracker recognizing physical and virtual representations of a head phantom.<br>Connected with a foot pedal to interact with the system and to toggle between AR and MR<br><br>(Not reported)	AR/MR	11.5	4
Aebersold et al. (2018)	Preliminary evaluation of a procedure training application<br><br>(Simulating nasogastric tube (NGT) insertion on phantom)	Mixed methods study: Randomized controlled trial (RCT) and survey<br><br>(69 nursing students, Control=34; AR=35)	Test:<br>1) Self-developed checklist for performance<br>Questionnaire:<br>2) Likert scale on LE	Statistically significant correct placement of NGT through all checklist items in the AR group vs. control (p<.011).<br>Participants' agreed /strongly agreed that AR was better for visualization (p<.01) and useful as tool in skill training (p<.015)	Company-developed application for mobile devices<br><br>(20-25 minutes)	AR	15.5	5

Albrecht, Folta-Schoofs, Behrends, & Von Jan (2013)	Comparative study of an application<br><br>(Learning of gunshot wounds)	Mixed methods study: RCT (pretest and post-test) and survey<br><br>(10 medical students, Control=4; AR=6)	Test (pre- and post-completion):<br>1) Self-developed single choice (improvement)<br>Questionnaire:<br>2) AttrakDiff2 (Likert scale) on LE<br>3) POMS on Mood States (pre- and post-completion)<br>Observation (by non-participants):<br>Directly on learning behavior	The test score was significantly improved in AR group (p<.03)<br>Hedonic quality was significantly favored by AR group (p<.005).<br>Fatigue and numbness significantly decreased, and vigor rose in the AR group.<br>Observations showed interactive discussion in AR group vs. individual approach in control group	Self-developed application for mobile devices recognizing markers overlaying images onto user's body<br><br>(30 minutes)	AR	14.5	4
Bifulco et al. (2014)	Investigation of the feasibility of an HMD-based application<br><br>(Recording an electrocardiogram (ECG) on phantom and healthy patient)	Two-group non-randomized comparison<br><br>(20 non-clinicians, manikin=10; patient=10)	Test:<br>1) Accuracy (average errors in mm)<br>2) Displacement errors (max error)	Average positioning errors of precordial electrodes were better on phantom vs. healthy patient. Max errors for the V6-lead <16 mm in both tests did not exceed clinical threshold of 25 mm	Self-developed for HMD with webcam recognizing markers attached to ECG device and phantom-patient<br><br>(Few minutes)	AR	10.5	3
Ferrer-Torregrosa, Torralba, Jimenez, García, & Barcia (2015)	Comparison of an application<br><br>(Learning anatomy of the lower limb)	Mixed methods study: RCT and survey<br><br>(211 students of anatomy, Control=134; AR=77)	Test:<br>1) Self-developed multiple choice<br>Questionnaire:<br>2) Self-developed on LE (metacognitive perception)	The AR group achieved significant better test result (p=.0001), and significantly surpassed the control group in terms of metacognitive perception (p<.05)	Self-developed for computer with webcam recognizing markers in printed book<br><br>(Not reported)	AR	15.5	4

Ferrer-Torregrosa et al. (2016)	Comparison of a didactic aid based on AR with images and video<br><br>(Learning anatomy of the foot muscles)	Mixed methods study: Three-group RCT and survey<br><br>(171 students of anatomy, images/ Control=60; Video=51; AR=60)	Test:<br><br>1) Self-developed Questionnaire:<br>2) Self-developed on LE (metacognitive perception)<br>3) Follow-up interview on learning success	Significant higher test score was obtained with aid of AR compared with video and notes (p<.000).<br>The metacognitive perception was significantly favored by the AR group (p<.05), also sharing higher expectations for AR-based learning success.	Company-developed for mobile devices recognizing markers in printed book<br><br>(14 days)	AR	13.5	4
Huang et al. (2018)	Investigation of the feasibility of an HMD-based application<br><br>(Simulating US-guided CVC on phantom)	Mixed methods study: Prospective RCT and survey<br><br>(32 novice operators, Control=16; AR=16)	Test:<br><br>1) Cannulation time<br>2) Procedure time<br>3) Adherence level<br>Questionnaire:<br>4) Expert-developed on LE (usability and ergonomics)	No significant difference in cannulation time (p=.09) or procedure time (p=.29) for the AR group vs. Control.<br>Adherence level were significantly favored by the AR group (p=.003).<br>The majority >80% accepted the device in terms of ergonomics.	Self-developed for HMD rendering an instructional slide show connected to a computer and a foot pedal to navigate between the content<br><br>(5-10 minutes)	AR	13.5	5
Jeon, Choi, & Kim (2014)	Investigation of a novel visualization device<br><br>(Simulating US-guided CVC on phantom)	Prospective cross-over trial<br><br>(20 physicians, Control/AR=20)	Test:<br><br>1) Time<br>2) No. needle redirections	Median of procedure time was clinically significant reduced by 50% in AR group vs. Control (p<.001). The number of needle-redirections significantly decreased in the AR group (p<.001)	Self-developed for micro projector attached to an ultrasound probe projecting images directly onto phantom<br><br>(10 minutes)	AR	11.5	2

Keri et al. (2015)	Evaluation of a needle guidance system<br><br>(Simulating lumbar puncture on phantom with abnormal spine)	RCT<br><br>(24 residents, Control=12; MR=12)	Test<br>(without assistive MR):<br>1) Needle path<br>2) Tissue damage<br>3) Procedure time<br>4) Needle insertion time<br>5) Success rate	Residents trained with MR visualization had better performance metrics: The MR group outperformed the control group significantly for needle path (p=.02), tissue damage (p=.01) and needle insertion time (p=.05) but not procedure time (p=.06) or success rate (p=.99)	Company-developed for computer, ultrasound machine, and tracker sensor-recognizing a virtual model of a vertebral column registered to a physical phantom<br><br>(20 minutes)	MR	12.5	5
Kugelmann et al. (2018)	Evaluation of the feasibility of a tutorial<br><br>(Learning of human gross anatomy)	Prospective large-scale cross-over survey<br><br>(880 medical students, Control/AR=880 /748 in survey)	Questionnaire:<br>1) Likert scale on LE<br>2) Advantages and disadvantages<br>3) 4-item rating of the tutorial	The students agreed that the system increased the motivation 59% and greatly improved 3D understanding 93.4% (strongly agreed).<br>AR was found advantageous to traditional books and rated ‘good’ by 81.9%	Company-developed for a computer connected to two cameras recognizing sensor-landmarks and overlaying images onto user’s body<br><br>(Before/during the tutorial)	AR	7	2
Küçük, Kapakin, & Göktaş (2016)	Determination of learning effect via mobile AR<br><br>(Learning of neuroanatomical pathways)	Mixed methods study: RCT and survey<br><br>(70 medical students, Control=36; AR=34)	Test:<br>1) Self-developed multiple choice<br>2) Self-translated Cognitive Load (Likert) Scale<br>Questionnaire:<br>3) Interview on LE	Achievement was significantly higher (p<.05) and cognitive load significantly lower reported in AR group (p<.05).<br>Of students in AR group 79% responded that mobile AR facilitated learning the subject	Company-developed for mobile devices recognizing markers in printed book<br><br>(5 hour-course)	AR	14.5	5

Leitritz et al. (2014)	Evaluation of the usability of an HMD-based application for examination<br><br>(Training ophthalmoscopy on head phantom and test person)	Mixed methods study: RCT and survey<br><br>(37 medical students, Control=18; AR=19)	Test:<br>1) Accuracy (No. of sketched vessels)<br>2) Self-developed (OTS) score<br>Questionnaire:<br>3) Likert scale on LE (self-evaluation)	Significantly higher accuracy (p<.0083) and OTS vs. Control (p<.0033), but self-evaluation was not significantly different between the two groups	Company-developed for HMD connected to computer recognizing a model lens and a head phantom<br><br>(15 minutes)	AR	14.5	4
Ma et al. (2016)	Investigation of precision of a personalized system<br><br>(Learning of human gross anatomy)	Two single-group posttests and survey<br>(Study 1)<br>(2 surgeons and 5 medical students)<br>(Study 2)<br>(72 medical students)	Test (quantified by participants):<br>1) Accuracy<br>(Study 1)<br>Questionnaire:<br>2) Likert scale on usability<br>3) Likert scale on LE<br>(Study 2)	Accuracy was demonstrated, and study participants favored the usability.<br>The learning potential of AR was accepted by 86.1%, and found valuable as a display system of anatomy 91.7%	Company-developed for computer connected to two cameras<br>recognizing sensor-landmarks and overlaying images onto user's body<br><br>(15 minutes)	AR	7.5	2
Mewes et al. (2019)	Provision and evaluation of a needle guidance system<br><br>(Simulating MR-guided needle insertion into calibration phantom)	Single-group posttest and survey<br><br>(4 radiologists and 4 technicians)	Test:<br>1) Entry point error<br>2) Target point error<br>3) Insertion time<br>Questionnaire:<br>Expert-interview on LE (usability)	The targets were reached, and the answers of the users were predominantly positive supporting the suitability of the system	Self-developed for projector coupled to two cameras inside a wide-bore MRI scanner recognizing markers on phantom<br><br>(Until users felt confident)	AR	10.5	3
Moro, Štromberga, Raikos, & Stirling (2017)	Comparison of an AR module with two learning modes (virtual reality (VR) and tablet)<br><br>(Learning of skull anatomy)	Mixed methods study: Three-group RCT and survey<br><br>(59 health science students, tablet/Control=22; VR=20; AR=17)	Test:<br>1) Self-developed multiple choice<br>Questionnaire:<br>2) Scale on adverse health effects<br>3) Likert scale on LE	No significant difference in test scores between the three learning modes (p<.874).<br>Adverse effects as dizziness were significantly experienced in the VR group vs. AR and tablet group (p<.001).<br>Perception of AR was high but not significant	Self-developed for mobile devices<br><br>(10 minutes)	AR	13.5	5

Moult et al. (2013)	Evaluation of a needle guidance system<br><br>(Simulating diagnostic US-guided facet joint injections on phantom)	RCT<br><br>(26 pre-medical undergraduate students, Control=13; MR=13)	Test (without assistive technology):<br>1) Success rate<br>2)Total time<br>3) Time inside<br>4) Total path<br>5) Path inside	Significantly higher mean success rate of 61.5% in MR group vs. Control 38.5% (p=.031). No significant difference was found in any of the needle metrics of procedure times or path lengths	Company-developed for computer, ultrasound machine, and tracker sensor-recognizing a virtual model of a vertebral column registered to a physical phantom.<br><br>(10 minutes)	MR	13.5	4
Noll, Von Jan, Raap, Albrecht, & Albrecht (2017)	Comparison of an AR application with mobile blended learning environment<br><br>(Diagnosing various skin diseases)	Mixed methods study: RCT (pretest, posttest, follow-up) and survey<br><br>(44 medical students, mobile phone/Control=22; AR=22)	Test (pre-, post- and follow-up-completion):<br>1) Self-developed single choice (improvement)<br>2) Retention (average decrease of correct answers)<br>Questionnaire:<br>3) AttrakDiff2 on LE<br>4) POMS on Mood States (pre- and post-completion)	No significant difference in test score or retention of knowledge.<br>No significant variations were found regarding experience and emotions between the groups of AR and mobile blended learning	Self-developed application for mobile devices recognizing markers overlaying images onto user's body<br><br>(45 minutes)	AR	14.5	6
Rai, Rai, Mavrikakis, & Lam (2017)	Validation and assessment of the efficacy of an HMD-based application<br><br>(Training ophthalmoscopy on head phantom)	Prospective three-group RCT<br><br>(28 novice residents and 3 fellows (experts), Control=15; AR=13; No training=3 (experts))	Test:<br>1) Total time<br>2) Total score<br>3) Performance (task scores/time)	Time required was not significantly different (p=.11), but the AR group significantly demonstrated superiority in total score (p=.02) and performance (p=.006). Fellows outperformed novice residents despite no prior experience with simulator	Company-developed for HMD connected to computer recognizing a model lens and a head phantom<br><br>(About 2 hours)	AR	14.5	5
Robinson et al. (2014)	Evaluation of a new MR part-task trainer	Mixed methods study: Three-group non-randomized comparison and survey	Test (pre- and post-intervention without assistive technology):<br>1) SCVA score	All participants significantly improved SCVA score (p<.0001) and time (p<.0001). The participants	Self-developed for computer with tracker sensor-recognizing a virtual model of the phantom registered within a	MR	13.5	7

	(Simulating subclavian venous access (SCVA/CVC) without US-guidance on phantom)	(65 physicians of different training categories, novices=25; intermediates=24; experts=16)	2) Time<br>3) No. attempts<br>4) No. skin punctures<br>5) Success rate<br>6) Complication rates (pneumothorax and subclavian puncture)<br>Questionnaire:<br>5) Likert scale on LE (usability)<br>6) Likert scale on performance confidence (pre- and post-intervention)	significantly reduced no. attempts (p<.0001), no. skin punctures (p=.0007), but no significant difference was found though success rate was increased (p=.08). Both complication rates fell with MR.<br>The majority 95.4% strongly agreed the usability for future CVC.<br>Confidence significantly rose (p<.0001)	3D-printed phantom built-up of head and thorax CT scan<br><br>(Until users felt confident)			
Rochlen, Levine, & Tait (2017)	Evaluation of usability of an HMD-based needle guidance system<br><br>(Simulating CVC without US-guidance on phantom)	Mixed methods study: Two-group non-randomized comparison and survey<br><br>(40 medical students /participants,<br>No prior CVC training=13; prior CVC training=27)	Test:<br><br>1) Correct identification<br>2) Correct needle insertion (accuracy)<br>3) Time<br>Questionnaire:<br>4) Likert scale on LE<br>5) Open-ended evaluation (ergonomics)	No significant difference in identification, needle insertion, and time expense between experienced and non-experienced.<br>Participants favored AR in visualizing anatomy 92.5% and for incorporation into training 82.1%.<br>Evaluation addressed issues of poor ergonomics <44.4%	Self-developed for HMD with external camera recognizing markers on needle and phantom<br><br>(Until users felt confident)	AR	14	3
Siebert et al. (2017)	Comparative investigation of adherence to a guideline adapted for HMD<br><br>(Simulating pediatric cardiopulmonary resuscitation on phantom)	Mixed methods study: Prospective RCT and survey<br><br>(20 residents, pocket reference cards/Control=10; AR=10)	Test (deviation from guidelines):<br><br>1) Time to first defibrillation/DF<br>2) Time to first compression<br>3) Drug and shock doses<br>4) No. of shocks<br>Questionnaire:<br>5) Likert scale on LE (stress perception)	Adherence by time to first DF and compressions were not improved, but errors were significantly reduced in administering shock doses vs. Control (p<.001).<br>No significant difference in stress response (p=.38)	Self-developed for HMD rendering guideline cards in the glasses with touchpad to navigate between the content<br><br>(15 minutes)	AR	13.5	6

Solbiati et al. (2018)	Preliminary assessment of a needle guidance system<br><br>(Simulation CT scan-guided needle insertion into phantom, porcine, and cadaver)	Single group posttest (proof-of-concept study)<br><br>(Study participants not specified)	Test:<br>1) Computed accuracy (mm)	An acknowledged targeting accuracy was achieved in all cases but in the breathing porcine model	Self-developed for mobile devices recognizing markers on tool and phantom-porcine-cadaver.<br><br>(Not reported)	AR	8.5	2
Sutherland, Hashtrudi-Zaad, Sellens, Abolmaesumi, & Mousavi (2013)	Demonstration of the potential and functionality of an application<br><br>(Simulating US-guided spinal needle insertion on phantom)	Two-group non-randomized comparative survey<br><br>(10 participants, residents=4; students and technicians=6)	Test:<br>1) Force (traversing of tissue)<br>Questionnaire:<br>1) Likert scale on LE (functionality)	Peak values of the forces and the pattern of the profile corresponded to related work. The system was positively reviewed on the system regarding functionality, visual feedback, and haptic feedback	Self-developed for computer coupled to a haptic device with stylus and camera recognizing sensors attached to a dummy ultrasound probe and a phantom.<br><br>(5-10 minutes)	AR	9.5	2
L. L. Wang, Wu, Bilici, & Tenney-Soeiro (2016)	Implementation and demonstration of a prototype<br><br>(Test preparation for neurologic clinical shelf exam)	Single-group survey<br><br>(24 medical students)	Questionnaire:<br>1) Query of LE (utility)	Upon demonstration 100% of participants agreed that AR improved the learning capacity for the textbook	Self-developed for mobile devices recognizing markers in printed book<br><br>(Demonstration)	AR	7	1
Wang et al. (2017)	Evaluation of feasibility and user experience of an HMD-based telemedicine mentoring platform<br><br>(Training US examination for trauma on healthy patient under guidance of mentor)	Three-group non-randomized comparison and survey<br><br>(24 medical students and 1 mentor,<br>Full telemedicine setup/Control=12; AR=12; mentor=1)	Test:<br>1) Expert-Global Rating Scale for performance<br>2) Completion time<br>Questionnaire:<br>3) Likert scale on LE (utility)<br>4) Cognitive load	Performance of the AR group was not significantly improved (p=.534), but the AR group had a significant prolonged completion time (p=.008).<br>The AR group showed no significant difference though they favored the utility of AR (p=.065) and reported a lower cognitive load (p=.28)	Self-developed for HMD with an ultrasound probe connected to computer and live-streamed to mentor connected to a sensor-controller projecting mentor's hands and gestures back into the AR space of the trainees<br><br>(No prior training)	AR	12	7

Zhu, Fors, & Smedberg (2018)	Exploration of needs and challenges in applying AR in continuing professional development (CPD)<br><br>(Training of general practitioners within primary care in China)	Qualitative semi-structured face-to-face interviews<br><br>(13 physicians and 2 managers)	Questionnaire:<br>1) Interview on attitudes toward usage<br>2) Query of suitability for subjects in future	The participants reacted positively to usage of AR in CPD, especially concerning visualization and skill training.<br>The design should improve competencies, understand learning needs, and stimulate positive attitudes toward technology	Company-developed application for mobile devices<br><br>(Demonstration)	AR	12 (AQRAME) (12)	6

KEY: HMD, head-mounted display; AR, augmented reality; MR, mixed reality; LE, learning experience; CVC, central venous catheterization; US, ultrasound
