# Supplementary material for: Augmented reality and mixed reality for healthcare education beyond surgery: an integrative review
Source: Int J Med Educ. 2020 Jan 18;11:1–18. doi: 10.5116/ijme.5e01.eb1a (PMC7246121; doi:10.5116/ijme.5e01.eb1a)
Supplement: Supplementary file 2 — Appendix 2. Distribution of studies across medical specialty or health science, number of studies, and participants enrolled according to number of studies [file ijme-11-1-S2.pdf]

Appendix 2.

Distribution of studies across medical specialty or health science, number of studies, and participants enrolled according to number of studies

Medical Specialty or Health Science	Subjects of Healthcare Education	No. Studies	No. Participants (according to number of studies)
Anatomy (6 studies)	Foot Muscles<br>Lower Limb<br>Skull<br>Human Gross Anatomy<br>Neuroanatomical Pathways	1<br>1<br>1<br>2<br>1	171<br>211<br>59<br>880+72<br>70
Anesthesia (7 studies)	Central Vein Catheterization<br>Lumbar Puncture<br>Spinal Needle Insertion<br>Ultrasound Examination for Trauma (Telemedicine)	4<br>1<br>1<br>1	32+20+65+40<br>24<br>10<br>24
Cardiology	Electrocardiogram Recording	1	20
Dermatology	Skin Diseases	1	44
Family Medicine	Continuing Professional Development*	1	15
Forensic Medicine	Gunshot Wounds	1	10
Gastroenterology	Nasogastric Tube Insertion (Nursing)	1	69
Neurology	Shelf Exam Preparation	1	24
Ophthalmology (2 studies)	Binocular Indirect Ophtalmoscopy	2	37+31
Orthopedics	Facet Joint Injections	1	26
Pediatrics	Cardiopulmonary Resuscitation	1	20
Radiology (3 studies)	Resection Planning (Neurosurgery)<br>Needle Insertion (MRI)<br>Needle Insertion (CT scan)	1<br>1<br>1	21<br>8<br>Not Specified

*A theoretical application was devised from an anatomy aplication.[47]
